# Management of skeletal Cl III malocclusion using simultaneous alternate rapid maxillary expansion and constriction (Alt-RAMEC) and facemask protraction in adolescence

**DOI:** 10.34172/joddd.2022.010

**Published:** 2022-05-29

**Authors:** Harpreet Singh, Pranav Kapoor, Poonam Sharma, Raj Kumar Maurya, Tanmay Mittal

**Affiliations:** ^1^Department of Orthodontics and Dentofacial Orthopedics, ESIC Dental College and Hospital, Delhi, India; ^2^Department of Dentistry, Central Government Dental Unit, Dimapur, Nagaland

**Keywords:** Adolescence, Alt-RAMEC, Cl III malocclusion, Orthopedic, Protraction facemask, Stability

## Abstract

Cl III malocclusion with a significant skeletal component presents a therapeutic challenge during adolescence. This article presents the encouraging results of an individualized two-stage treatment approach adopted for successful nonsurgical correction of severe skeletal Cl III malocclusion in an adolescent girl after the onset of puberty. An orthopedic approach involving simultaneous alternate rapid maxillary expansion and constriction (Alt-RAMEC) protocol and protraction facemask (PFM) therapy was adopted in phase 1 to correct the sagittal skeletal discrepancy. In phase 2, fixed orthodontic therapy aided by the interim use of a modified occlusal settling appliance was undertaken to obtain well-interdigitated occlusion. Meticulously planned and well-executed orthopedic and orthodontic approach, combined with good patient compliance and favorable growth pattern, helped establish well-balanced facial harmony with a proper maxillomandibular relationship and satisfactory overjet and overbite. The results remained stable during the 4-year follow-up. Alt-RAMEC-PFM therapy accompanied by fixed mechanotherapy is a viable option to treat severe skeletal Cl III malocclusion in adolescents.

## Introduction

 CI III malocclusion, although less prevalent than Cl I and Cl II malocclusion,^1^usually poses a complex therapeutic challenge. Since late mandibular growth tends to worsen the Cl III discrepancy,^2^optimizing treatment timing is crucial for ensuring successful management as treatment difficulty increases considerably over time. During growth, maxillary deficiency has been documented to be a significant etiologic component^2,3^;it has also been reported to be a key determinant for good prognosis.^4,5^

 Rapid maxillary expansion (RME) accompanied by protraction facemask (PFM) therapy is a popular modality for early correction of skeletal Cl III malocclusion during the deciduous and mixed dentition periods.^6-8^More recently, early treatment with Liou’s^9^ alternate RME and constriction (Alt-RAMEC) protocol has been demonstrated to enhance subsequent sagittal maxillary protraction, albeit on a short-term basis.^10^However, there is limited literature on the long-term positive response to concurrent Alt-RAMEC and PFM therapy to correct severe skeletal Cl III malocclusion in adolescents to the best of our knowledge.

 This article reports the effectiveness of an individualized orthopedic‒orthodontic approach adopted for successful treatment of an adolescent girl with severe skeletal Cl III malocclusion. The treatment involved concurrent Alt-RAMEC and PFM therapy as the initial first stage orthopedic procedure, accompanied by comprehensive fixed mechanotherapy performed during the second stage. The stability of the clinically acceptable aesthetic and functional outcomes are also discussed.

## Case Presentation

**Figure 1:**
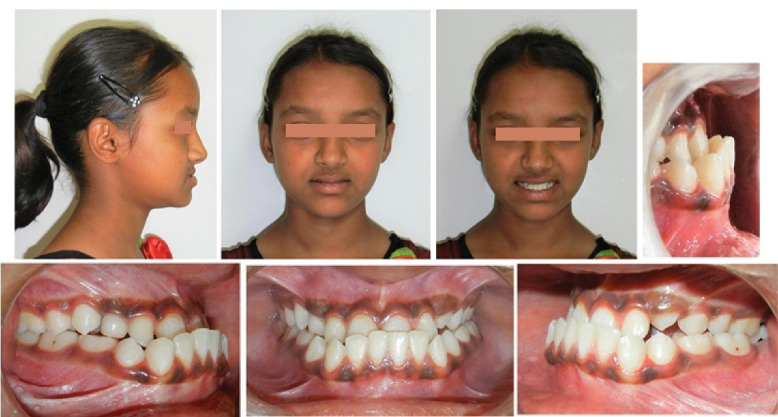
Pretreatment extraoral and intraoral photographs showing Cl III malocclusion due to maxillary retrognathism and mandibular prognathism.

 A 12-year-old girl presented, complaining of prominent lower front teeth, protruded chin, and compromised mastication and phonation. She had very low self-esteem as she was constantly ridiculed for her unpleasant smile and facial appearance. The family history was not suggestive of any genetic predisposition. Extraoral examination revealed a bilaterally symmetrical face, a mesoprosopic facial form, a concave facial profile with midface deficiency, a retrusive upper lip, a prominent lower lip, an acute nasolabial angle, and a shallow mentolabial sulcus (Figure 1). A low lip line was evident upon smiling with almost negligible maxillary incisor display and excessive mandibular incisor display. CO-CR discrepancy was discernible with the inability to move the mandible backward with the incisors edge to edge in the retruded contact position. However, mild deviation of the mandible towards the left was noted on jaw closure from initial contact position to habitual occlusion position. The tongue was normal in size and function. In addition, the patient exhibited habitual oral breathing despite a patent nasal airway. TMJ exhibited normal functional activity.

 Intraorally, she displayed bilateral Cl III molar and canine relationships with retained upper right deciduous canine and lower deciduous second molars. A long span of crossbite extending from the maxillary right deciduous canine to the left permanent first molar was observed. Incisor overbite was 5 mm, and overjet was -4 mm. The upper dental midline was shifted to the right of the facial midline by 2 mm. The lower dental midline was shifted to the left by 3mm compared to the upper dental midline (Figure 1). Both dental arches were relatively well-aligned. Model analysis revealed a transpalatal arch width of 33 mm in the first molars.

**Figure 2:**
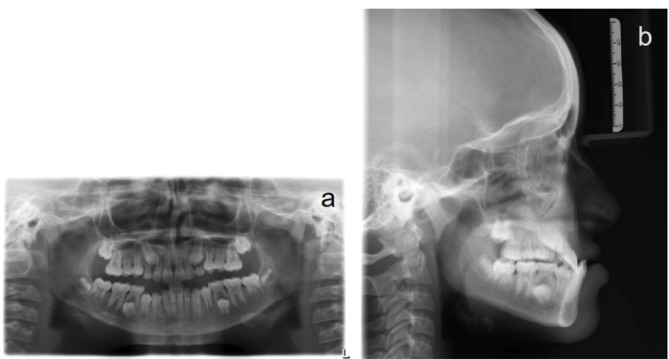
Pretreatment radiographs. (a) Panoramic radiograph, (b) Lateral cephalogram.

 A panoramic radiograph, taken at the early permanent dentition stage of development, did not reveal any bony or periodontal abnormalities (Figure 2a). Cephalometric analysis revealed a skeletal Cl III anteroposterior relationship (ANB, -6°; Wits appraisal, -8 mm), a retrognathic maxilla (SNA, 76°), a prognathic mandible (SNB, 82°), and a hypodivergent growth pattern. A large maxillomandibular discrepancy with Co-A to Co-Gn of 26 mm was noted (the normal range is 20‒23 mm). The maxillary incisors were slightly proclined (U1 to SN, 105°), whereas mandibular incisors had normal inclinations (IMPA, 89°) (Figure 2b; Table 1). The soft tissue analysis confirmed upper lip retrusion and lower lip protrusion. The patient was in the CS3 stage of skeletal maturation, according to the CVMI method.

**Table 1:** Lateral Cephalometric Analysis

**Variable**	**Pretreatment**	**Posttreatment**	**4-year follow-up**
**Sagittal**			
SNA (˚)	76	79	79
SNB (˚)	82	78	79
SND (˚)	79	77	77
ANB (˚)	-6	1	0
Wits (mm)	-8	-1	-1
Co-A (mm)	81	89	90
Co-Gn (mm)	107	119	120
**Vertical**			
SN-GoGn (˚)	28	32	31
FMA (˚)	19	24	23
SN- FH	11	12	12
ANS-Me/N-Me	0.529	0.558	0.549
**Dental**			
U1-SN (˚)	105	117	118
U1-FHP	115	126	128
U1 to NA (˚)	27	34	35
U1 to NA (mm)	5	7	8
L1 to NB (˚)	20	17	18
L1 to NB (mm)	3	3	3.5
IMPA (˚)	89	86	86
L1-APo (mm)	7	3	4
U1-L1 (˚)	138	140	129
**Soft tissue**			
H line-nose (mm)	+10	+ 9	+10
Upper lip-S line (mm)	-2	0	-1
Lower lip- S line (mm)	+4.5	0	0
Upper lip protrusion-esthetic plane (mm)	-7	-4	-4
Lower lip protrusion- esthetic plane (mm)	+1	-2	-2
**Pharyngeal airway measurements**			
McNamara’s upper pharyngeal width (mm)	11	18	18
McNamara’s lower pharyngeal width (mm)	12	11	11

 Based on the clinical and cephalometric examinations, the cause of this patient’s malocclusion was an underdeveloped maxilla and mandibular prognathism.

###  Treatment objectives

 The treatment goals were to (1) improve the skeletal jaw relationship by protracting the maxilla anteriorly relative to the cranium and redirecting mandibular growth, (2) correct anterior and posterior crossbites, (3) eliminate CO/CR discrepancy, (4) achieve esthetically favorable and functionally effective overjet and overbite, (5) establish canine-guided functional occlusion with anterior guidance, (6) improve frontal and profile esthetics, and (7) ensure that the patient breathed mostly through the nose.

###  Treatment plan and alternatives

 Considering the patient’s age and severity of the skeletal and occlusal disharmony, a bone-anchored maxillary protraction treatment protocol was suggested since it offers the advantages of ensuring 24-hour bone-borne force, at the same time avoiding the problems associated with dental anchorage. However, the patient and her parents declined this approach because of the underlying concerns about the associated surgical risks. Thus, a conservative two-stage treatment approach commensurate with the patient’s young age and the parents’ wishes was adopted. An orthopedic approach involving concurrent Alt-RAMEC and PFM therapy for effective maxillary advancement was contemplated as a stage 1 procedure, followed by fixed orthodontic mechanotherapy during phase 2 to achieve well-interdigitated buccal occlusion. However, the parents were informed of the possible need for orthognathic surgery in the future, considering the likelihood of intermaxillary sagittal relationship worsening if the craniofacial growth proved unfavorable or if the patient did not comply with the recommended duration of orthopedic treatment.

###  Treatment progress

**Figure 3:**
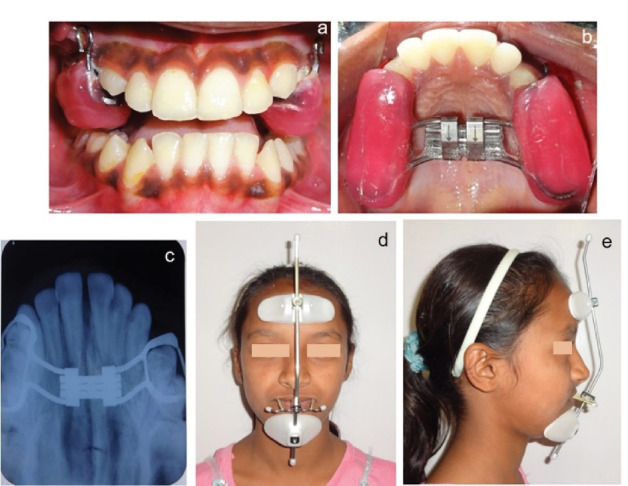
Treatment progress photographs. (a) Hyrax expander with soldered hooks for the facemask. (b) Maxillary occlusal view with bonded Hyrax assembly. (c) Post-Alt-RAMEC maxillary occlusal radiograph showing midpalatal suture opening. (d) Frontal photograph with facemask.(e) Profile photograph with facemask.

 The first phase of treatment involved the Alt-RAMEC protocol performed with an 11-mm bonded Hyrax-type expander (Leone A2620, Leone Orthodontic Products, Italy), with the simultaneous use of a Petit-type facemask for protraction of the maxilla. A 7-week Alt-RAMEC protocol of alternating expansions and constrictions commenced with expansion in the first week, alternating to constriction in the second week, and completed with expansion in the seventh week (Figures 3a, 3b, and 3c). Daily activation of the expansion or constriction was 0.4 mm (0.20 mm per turn, one turn in the morning, and one turn at night). 5/16”, 14 oz. Protraction elastics (600 g per side) were attached from the hooks on the maxillary expander near the maxillary canines, with a downward and forward pull of 30° from the occlusal plane (Figures 3d and 3e). The facemask was worn for 16 hours per day for nine months.

**Figure 4:**
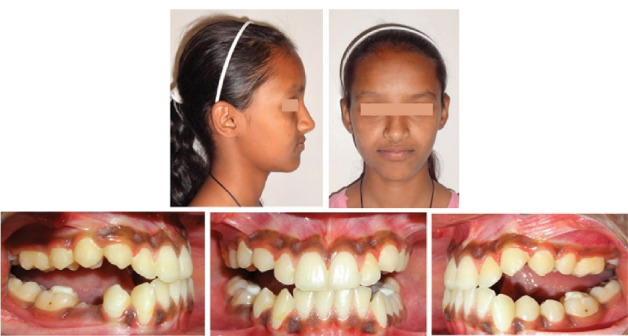
Post-orthopedic extraoral and intraoral photographs showing improvements in profile and appearance of bilateral posterior open bite.

**Figure 5:**
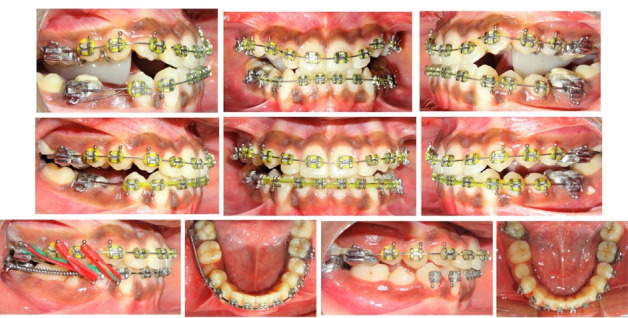
Stage photographs showing modified interim occlusal settling appliance, progression of leveling, and spontaneous eruption of mandibular right second premolar following space regaining, using compressed NiTi open-coil spring.

 Following the removal of bonded Hyrax assembly after six months, the skeletofacial esthetics improved considerably. However, a posterior open bite of large magnitude (approximately 5 mm) was observed (Figure 4). At the beginning of phase 2 treatment, 0.022*0.028-inch slot pre-adjusted fixed appliances (MBT prescription) were placed in the maxillary and mandibular dental arches. Initial alignment and leveling were achieved with sectional archwires in the upper arch and continuous wires in the lower arch, starting with improved superelastic 0.016-inch NiTi wire, followed by 0.020*0.020-inch SS wire. A modified occlusal settling appliance,^11^consisting of retentive pin-head clasps and lateral acrylic flanges, was used as an interim adjunct to prevent lateral tongue thrust to facilitate rapid settling of buccal segment occlusion (Figure 5).

 Third molar germectomy was performed, followed by distalization of the mandibular right second molar, using compressed nickel-titanium open-coil spring on a 0.017*0.025-inch stainless steel (SS) wire to create space for mandibular right second premolar. Simultaneous use of short Cl III elastics helped counteract the mesial reactionary forces. Once adequate space had been created, the second premolar erupted spontaneously in its position in the arch (Figure 5). A normal sequence of continuous stainless-steel archwires was subsequently used to level and coordinate the arches.

 While maintaining the original intercanine width in mandibular arch form, individualized first- and third-order bends were carefully incorporated in the maxillary and mandibular continuous 0.019*0.025-inch SS archwires to detail the tooth positions. Judicious use of short and light (3.5 oz.) Cl III and vertical spaghetti elastics helped improve occlusal interdigitation.

 After debonding, retention consisted of maxillary and mandibular wraparound Hawley retainers worn during the daytime and a reverse twin block (RTB) appliance at night for 24 months. The patient was regularly monitored every six months to evaluate mandibular growth.

###  Treatment results

**Figure 6:**
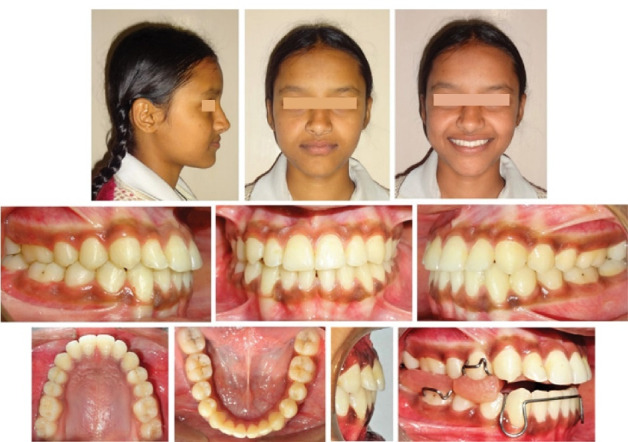
Post-treatment extraoral and intraoral photographs showing restoration of facial esthetics with well-intercuspated Cl I occlusion and use of RTB during the retention phase.

 The total treatment duration was 27 months. Post-treatment final records showed the achievement of the desired treatment objectives, i.e., significant improvement in facial esthetics with the correction of the maxillary deficiency, better lip support and an improved nasolabial angle, along with the establishment of normal overjet and overbite, and well-intercuspated buccal occlusion with canine guidance (Figure 6). Dramatic improvement of the lip line and smile arc was discernible with adequate maxillary incisor display on smiling. Anterior movement of the upper lip, posterior movement of the lower lip, and soft tissue pogonion contributed to improvements in the patient profile. Nasal breathing also showed spontaneous improvements. Reverse twin block with upper and lower acrylic guided components was used as passive retention at night to prevent relapse (Figure 6).

**Figure 7:**
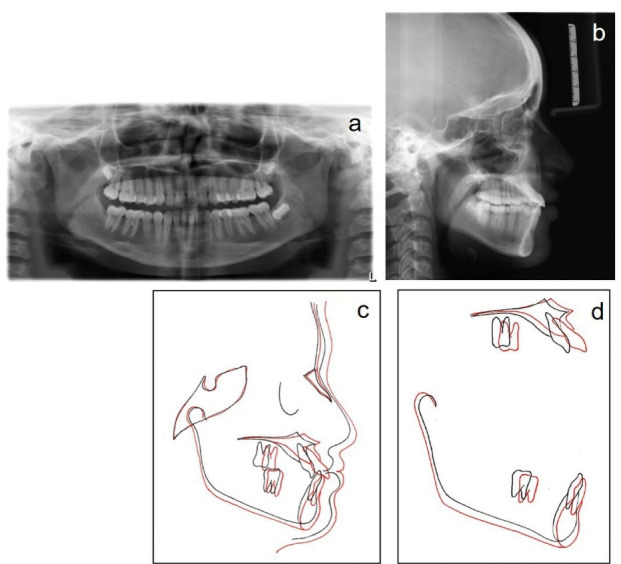
Post-treatment radiographs. (a) Panoramic radiograph. (b) Lateral cephalogram. (c) Overall superimposition registered on sella. (d) Regional (best-fit) superimpositions in the maxilla and mandible.

 A panoramic radiograph revealed good root parallelism with no significant apical resorption (Figure 7a). Cephalometric superimposition demonstrated improvements in the maxillomandibular relationship (Wits, −8 mm→ -1 mm; ANB, -6° → 1°), the sagittal position of the maxilla (SNA, 76° →79°), and establishment of a harmonious soft-tissue profile along with the clockwise rotation of mandibular base (FMA, 24°; SN-GoGn, 32°) (Figures 7b, 7c, and 7d).

**Figure 8:**
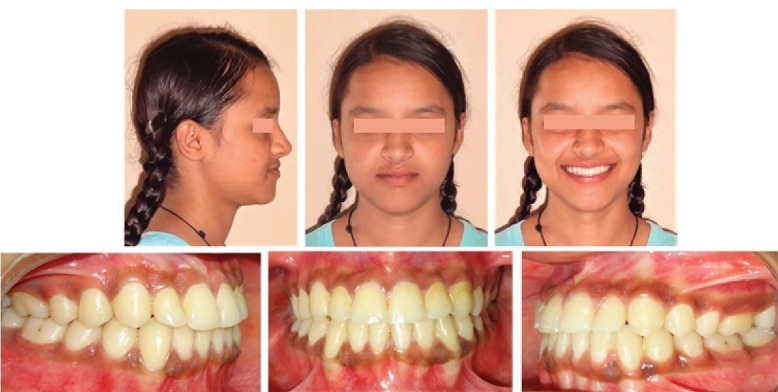
2.5-year follow-up demonstrating the stability of achieved results.

**Figure 9:**
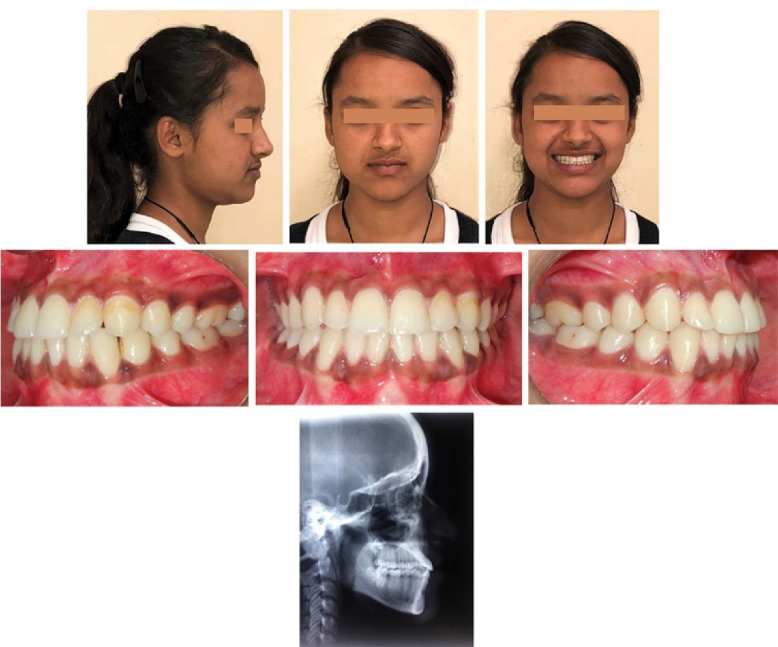
Clinically and radiographically stable results at 4-year follow-up.

 At 2.5-year and 4-year follow-up appointments, the harmonious facial balance and intermaxillary dental relationships were well-maintained (Figures 8 and 9).

## Discussion

 Treating severe skeletal Cl III malocclusion during adolescence is challenging, especially when a conservative approach is adopted. It is imperative that the selected treatment plan reflect optimal treatment timing and prognosis of growth by evaluating skeletal maturity.^12^With CVMI indicating 25%-45% of the remaining skeletal growth, and considering the small window of nonsurgical therapeutic opportunity due to the possibility of heavy interdigitation of the circummaxillary sutures around puberty,^13^it was decided to perform PFM therapy simultaneously with the Alt-RAMEC procedure, rather than waiting until the completion of the Alt-RAMEC protocol. This modification was under the recommendations of Canturk and Celikoglu,^14^ who demonstrated similar positive outcomes with this modified orthopedic protocol.

 Alt-RAMEC was adopted as the procedure of choice instead of RME since our patient exhibited no pure transverse maxillary deficiency with an intermolar distance of 33 mm, which was higher than McNamara’s^15^ norm of 31 mm. This facilitated effective and pronounced reciprocal disarticulation of both the sagittal and coronally running circummaxillary sutures, without consequent over-expansion, augmenting the skeletal effects of maxillary protraction.^16,17^

 Different weekly sequences of Alt-RAMEC protocol varying from 4 to 9 weeks have been proposed in the literature. However, there is no clear consensus about which weekly sequence should be employed. Similar effects have been reported with nine weeks and seven weeks of Alt-RAMEC for the subsequent maxillary protraction.^9,17^ Therefore, to avoid the impending possibility of the risk of creating a jiggling effect in recurring weeks with the 9-week Alt-RAMEC when employed in permanent dentition,^18^a 7-week protocol was selected for our patient. Moreover, the implementation of at least 7-week Alt-RAMEC facilitates the greater quantitative opening of the coronally running circummaxillary sutures needed for more effective maxillary protraction than the 5-week protocol that does not elicit adequate opening of coronally running sutures.^17^More recently, it has also been reported that reduced buccal alveolar bone thickness of the expander’s anchor teeth observed immediately after the 7-week Alt-RAMEC protocol is within the scope of the initial alveolar thickness of the expander’s anchor teeth.^19^With judicious application of alternating rapid expansion/constriction course at a rate of 0.4‒0.5-mm activation per day instead of 0.8‒1 mm per day, no deleterious post-expansion effects were observed concerning root or bony dehiscence or compromised periodontal support of the anchor teeth, and the patient well tolerated the entire treatment process.

 The well-documented orthopedic effects (forward and downward movement of the maxilla, restriction and redirection of mandibular growth with concomitant downward and backward mandibular rotation) and orthodontic effects (proclination of the maxillary incisors and retroclination of the mandibular incisors) induced by maxillary protraction^6,8,20^ significantly contributed to improvements in maxillomandibular and intermaxillary dental relationships, respectively, with the restoration of profile esthetics in our patient. It has been widely reported that the effects of maxillary protraction are predominantly skeletal in younger children and mostly dentoalveolar after 10 years of age.^6-8,21^The clinically significant maxillary advancement observed after pubertal onset in our case could be attributed to the synergistic effects of combined Alt-RAMEC and PFM therapy, enabling enhancement of the orthopedic effects of a facemask by the Alt-RAMEC protocol, and vice versa. The downward displacement of the maxilla also helped optimize the patient’s lip line and improved upper incisor exposure. Since the patient had a hypodivergent growth pattern, mild clockwise mandibular rotation did not compromise our patient’s esthetics. Moreover, according to the findings of Celikoglu and Buyukcavus,^22^significant improvements in upper pharyngeal airway dimensions and insignificant changes in the lower pharyngeal dimensions were also discernible in our patient.

 Germectomy is a short and simple procedure involving the removal of a tooth that had formed one-third or less of its root.^23^It is noteworthy that second molar distalization following elective third molar germectomy provided adequate space for the spontaneous eruption of the mandibular right second premolar.

 Considering the severity and magnitude of posterior open bite following facemask therapy, application of heavy intermaxillary elastic traction was not feasible for the posterior leveling of the occlusal plane. Hence, the interim use of a modified occlusal settling appliance was considered more appropriate for aiding the closure of open bite. Placement of third-order bends in 0.019*0.025-inch SS wire during the finishing stage helped prevent further excessive buccal flaring of maxillary incisor crowns and allowed labial bodily movement.

 Based on Wits appraisal of -8mm (i.e., between 4 and 12mm), the severity of Cl III patient featured in the present study was labeled as the ‘yellow’ category.^24^Even so, the application of a combination of simultaneous Alt-RAMEC protocol and PFM therapy, accompanied by fixed mechanotherapy, contributed to establishing well-interdigitated Cl I dental occlusion without the loss of dental units while restoring facial esthetics to acceptable harmonious levels.

 Institution of early protraction treatment before 10 years of age contributes to adult stability in 73%‒75% of cases.^21,25^However, since our patient underwent orthopedic treatment after the commencement of puberty, it cannot be denied that the stability could have been jeopardized due to any impending residual growth.^7^Long-term appraisal of the treatment outcomes showed that maxillary changes and alterations in the sagittal position of the mandible were well-maintained at the end of active craniofacial growth.

 Good patient compliance in using removable appliances (including retainers) proved crucial for ensuring the stability of treatment results.

## Conclusion

 In aptly selected cases, a synergistic combination of concurrent Alt-RAMEC and PFM therapy, accompanied by comprehensive fixed mechanotherapy, can be a viable treatment modality to correct severe skeletal Cl III malocclusion after the onset of puberty. This minimally invasive treatment protocol met the patient’s esthetic expectations, thereby uplifting her self-esteem.

 The reasonably stable results observed four years after treatment completion indicate that individualized treatment planning, adherence to sound orthopedic and orthodontic biomechanical principles, favorable growth pattern, and ensuring absolute patient compliance all contribute to maintaining long-term results.

## Authors’ Contributions

 All authors have made substantive contributions to this manuscript, and all have reviewed the final paper before its submission.

## Funding

 None.

## Ethical Approval

 This case report complies with CARE guidelines for case reports, and written informed consent was taken from the patient’s parents for photo release.

## Competing Interests

 The authors declare no conflict of interests in connection with this article.
